# Signal Source Localization with Long-Term Observations in Distributed Angle-Only Sensors

**DOI:** 10.3390/s22249655

**Published:** 2022-12-09

**Authors:** Shenghua Zhou, Linhai Wang, Ran Liu, Yidi Chen, Xiaojun Peng, Xiaoyang Xie, Jian Yang, Shibo Gao, Xuehui Shao

**Affiliations:** 1 National Laboratory of Radar Signal Processing, Xidian University, Xi’an 710071, China; 2 China Academy of Launch Vehicle Technology, Beijing 100076, China; 3 Beijing Aerospace Automatic Control Institute, Beijing 100854, China

**Keywords:** passive sensor networks, signal localization, angle-only observations, accuracy analysis

## Abstract

Angle-only sensors cannot provide range information of targets and in order to determine accurate position of a signal source, one can connect distributed passive sensors with communication links and implement a fusion algorithm to estimate target position. To measure moving targets with sensors on moving platforms, most of existing algorithms resort to the filtering method. In this paper, we present two fusion algorithms to estimate both the position and velocity of moving target with distributed angle-only sensors in motion. The first algorithm is termed as the gross least square (LS) algorithm, which takes all observations from distributed sensors together to form an estimate of the position and velocity and thus needs a huge communication cost and a huge computation cost. The second algorithm is termed as the linear LS algorithm, which approximates locations of sensors, locations of targets, and angle-only measures for each sensor by linear models and thus does not need each local sensors to transmit raw data of angle-only observations, resulting in a lower communication cost between sensors and then a lower computation cost at the fusion center. Based on the second algorithm, a truncated LS algorithm, which estimates the target velocity through an average operation, is also presented. Numerical results indicate that the gross LS algorithm, without linear approximation operation, often benefits from more observations, whereas the linear LS algorithm and the truncated LS algorithm, both bear lower communication and computation costs, may endure performance loss if the observations are collected in a long period such that the linear approximation model becomes mismatch.

## 1. Introduction

For some passive sensors, such as infrared sensors, photoelectric sensors and cameras, they can detect targets by receiving electromagnetic signals. As they do not emit signals, they can probe targets in a stealth manner [1]. However, such sensors can measure only angles of signals and thus are termed as angle-only sensors subsequently. The signal position information, which is of great concern in many situations, cannot be obtained with a sensor. To determine the position of signal sources, one can connect distributed sensors with communication links and then estimate the position through a fusion algorithm. This is a hot topic in recent years and gains wide attentions of scholars in different fields [2,3,4,5].

In the 3-dimensional (3D) scenario, the angle information measured by each passive sensor includes the azimuth and elevation of the signal. From a mathematical perspective, each angle observation can be represented by a straight line passing the sensor and a target in space. If no error occurs in this process, all the lines will intersect in a point in space, which is the location of a signal source. In practice, both sensor location measures and angle measures are inevitably contaminated by measurement noises and then the lines may not intersect a point in space. However, as if the signals are from the same target, the lines will intersect in a small volume, whose center can be deemed as the location of a target. Following this concept, an angle-only positioning algorithm is presented in [6] and a closed-form solution is derived.

In real applications, if the targets of interest are static, or if the sampling frequency to the signals is too high in contrast to the velocities of possible targets, the algorithms can be developed under an assumption that the velocity of the target is static. The least squares (LS) algorithm is applied in the target position estimation based on angle-only measurements by linearizing the angle observation equations [7,8,9,10]. The intersection localization algorithm is obtained by considering that the straight lines formed by the angle observations will intersect in a small volume in space [11,12,13]. Real sensor often makes observations in an asynchronous manner, namely the observations are not obtained at the same instants. The stationary target assumption will also make the sensor synchronization problem easier, because we can totally drop the timing information of the observations. If the target is stationary, even if the sensors are moving, the straight lines formed by the angle measurements of multiple sensors at different times will converge to a small area near the target location. Therefore, in this scenario, one just needs to solve a target positioning algorithm in an asynchronous manner.

Once the target motion should be considered at different observation instants, target location estimation will face greater biases and then one has to take the target motion issue into consideration. Meanwhile, for moving targets, the observation instants should be taken into account and then as the distributed fusion algorithm should take instant information into account, the fusion algorithm becomes more complicated. There are mainly two strategies available so far. The first strategy is to use the filtering algorithms, such as the Kalman filter that can estimate target velocity through observations from different instants. In the target tracking theoretical framework, the angle-only observations can be described by a measurement equation, although it is heavily nonlinear. Therefore, a nonlinear filtering algorithm should be used [14,15]. For instance, the extended Kalman filtering (EKF) algorithm linearizes the angle measurement function through the first-order Taylor approximation, and then uses the standard Kalman filtering algorithm for the angle-only target tracking problem [16]. The cubature Kalman filter (CKF) [17], the unscented Kalman filter (UKF) [18], the pseudo linear Kalman filter (PLKF) [19,20], the particle filter [21] and a series of sigma-point based algorithms can also be used in the target tracking problem with distributed angle-only sensors.

Although the tracking algorithms have been widely used to estimate moving target positions, it requires the noise distribution parameters known a priori. It also faces the convergence problem if the initial state is set improperly [22,23]. In distributed sensor networks, if each observation undergoes a tracking process, the computation cost will also be high since the data amount of observations are often intensive in practice. Therefore, a good positioning algorithm should be implemented before filtering. For instance, in [15], short-term angle-only observations are fused by a distributed positioning algorithm, whose outputs are then processed by a tracking algorithm.

In the other strategy, the target position and velocity can be estimated together and then the result is valid in a longer period. In this case, the tracking operation can be performed in a longer period, so that the computation cost can be further reduced. However, if the velocity is estimated, more optimization variables are involved and then the optimization problem is more complicated. Meanwhile, in a distributed sensor configuration, the communication cost between the sensors may be high if all the observations are transmitted to a fusion center. In this paper, we study the distributed positioning of moving targets with distributed asynchronous angle-only sensors. We consider the scenario where multiple asynchronous passive sensors are linked with the fusion center through communication links. First, we formulate an algorithm, termed as gross LS algorithm, that takes all angle observations of multiple sensors together with their positions in certain period to estimate the position and velocity of the target. Different observations contribute different lines and with many lines available, both the target position and its velocity can be estimated. The classical LS algorithm is formulated such that the computation cost is reduced a lot.

Due to the huge amount of data, this algorithm still has high computational complexity and high communication cost. In order to reduce the communication and computation cost, we further present a distributed positioning algorithm, termed as linear LS algorithm, that can implement the fusion algorithm in a parallel computation manner. In detail, both positions and angle observations of local sensors are processed by LS operations, whose outputs are zero and first orders of the Taylor series of corresponding parameters. The outputs are then transmitted to a fusion center for which we derive a fusion algorithm to efficiently combine position and velocity estimates for a higher parameter estimation accuracy. The later algorithm can greatly compress the data rate from local sensors to the fusion center, such that the communication cost is greatly reduced. Meanwhile, local observations are represented by a few parameters and thus the fusion algorithm also needs a lower computation cost. The sensor location can be recorded asynchronously with the angle observations and thus can make the algorithm easier in applications. Meanwhile, a truncated LS algorithm, which replaces the velocity estimation of the linear LS algorithm by a simply average operation, is also presented.

Numerical results are obtained with distributed asynchronous angle-only sensors measuring a moving target with certain velocity. The convergence performance of both the algorithms are presented first, in order to examine the impact of the number of observations on the positioning performance. Then the impact of the linear approximation of position and angle measures on the estimation accuracy is analyzed. It will be found that the gross LS algorithm often benefits from more observations. However, although the linear LS algorithm and the truncated LS algorithm will perform good if the number of observations is small, as the number of observations increase, their performances will degrade, as a resulting of the linear model mismatching. The truncated LS algorithm will perform better in a short period than the linear LS algorithm but worse in a longer time. To an extreme, the estimation performance of the linear LS algorithm may deteriorate with more observations if the model mismatching is severe. We also verify that the linear LS algorithm has a lower communication cost in most situations and examine the performance loss due to inaccurate platform velocity estimates. Numerical angle distortion errors under the linear approximations are also analyzed.

## 2. Localization with Angle-Only Passive Sensors

### 2.1. Signal Model of Passive Observations

**Figure 1:**
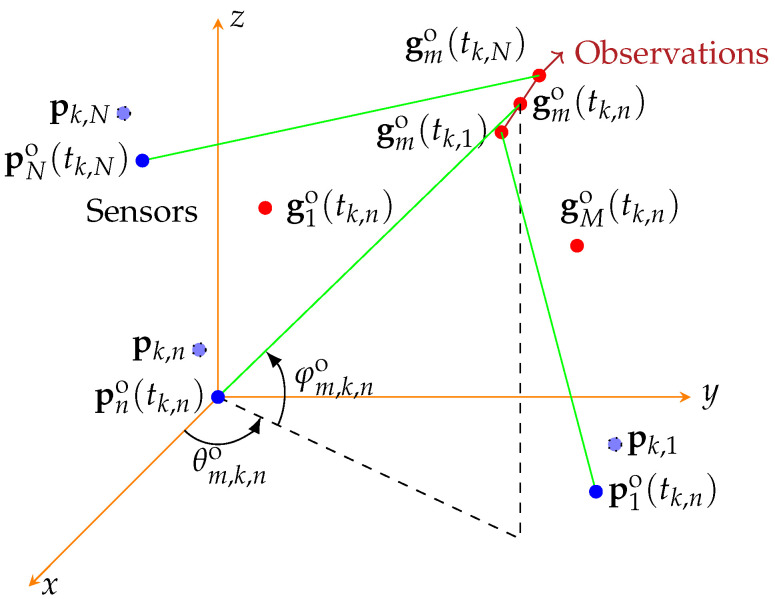
Measurement scenario of the passive sensors.

Consider a passive sensor network with *N* widely separated sensors and *M* targets in the surveillance volume. All the *N* passive sensors can measure only direction of arrival (DOA) of signals, based on which real position of a signal emitter can be estimated. Assume that all the sensors operate in the same coordinate system through some inherent position and attitude measurement devices, such as the Global Positioning System (GPS) and inertial sensors. A typical coordinate system is the earth-centered earth-fixed (ECEF) of the World Geodetic System 84 (WGS84). Both the targets and the sensors are in motion by assumption. The real position of the *n*th sensor at instant *t* is denoted by $p_{n}^{o}\left(t\right)={\left[x_{n,s}^{o}\left(t\right),y_{n,s}^{o}\left(t\right),z_{n,s}^{o}\left(t\right)\right]}^{T}$, n=1,2,…,N, where ${\left(\cdot \right)}^{T}$ denotes the transpose operation, and $x_{n,s}^{o}\left(t\right),y_{n,s}^{o}\left(t\right),z_{n,s}^{o}\left(t\right)$ denote the x,y,z coordinates of the *n*th sensor in the common coordinate system at instant *t*, respectively. The real position of the *m*th target at instant *t* is denoted by $g_{m}^{o}\left(t\right)={\left[x_{m,g}^{o}\left(t\right),y_{m,g}^{o}\left(t\right),z_{m,g}^{o}\left(t\right)\right]}^{T},m=1,\cdots ,M$, where $x_{m,g}^{o}\left(t\right),y_{m,g}^{o}\left(t\right),z_{m,g}^{o}\left(t\right)$ denote the x,y,z coordinates of the *m*th target at instant *t*, respectively. The topology of the passive sensors and targets are shown in Figure 1.

For the *n*th sensor, signals are detected and their DOAs are measured at instants denoted by $t_{k,n},k=1,\cdots ,N_{n}$, where $N_{n}$ denotes the number of observations of the *n*th sensor. At the instant $t_{k,n}$, assume that the position of the *n*th sensors is measured as

(1) $$p_{k,n}\left(t_{k,n}\right)=p_{n}^{o}\left(t_{k,n}\right)+\Delta p_{n}\left(t_{k,n}\right)={\left[x_{n,s}\left(t_{k,n}\right),y_{n,s}\left(t_{k,n}\right),z_{n,s}\left(t_{k,n}\right)\right]}^{T},k=1,\cdots ,N_{n}$$

where $\Delta p_{n}\left(t_{k,n}\right)$ denotes the sensor self-positioning error. For simplicity, we assume that the sensor self-positioning error follows zero mean Gaussian distributions with covariance matrices $C_{s}\left(k,n\right)=E\left(\Delta p_{n}\left(t_{k,n}\right)\Delta p_{n}^{T}\left(t_{k,n}\right)\right)$, where E denotes the expectation operation.

At instant $t_{k,n}$, the real position of the *m*th signal source is denoted by

(2) $$g_{m}^{o}\left(t_{k,n}\right)={\left[x_{m,g}^{o}\left(t_{k,n}\right),y_{m,g}^{o}\left(t_{k,n}\right),z_{m,g}^{o}\left(t_{k,n}\right)\right]}^{T},m=1,\cdots ,M.$$

Assume that all the observations regarding the same target are obtained in a short period $T=[T_{1},T_{2}]$. In this period, assume that the location of the *m*th target can be expressed by

(3) $$g_{m}^{o}\left(t\right)\approx g_{m}^{o}\left(t_{0}\right)+v_{m}^{o}\left(t-t_{0}\right)$$

where $g_{m}^{o}\left(t_{0}\right)$ denotes the location of the target at the reference instant $t_{0}$, and $v_{m}^{o}$ denotes the velocity over T. The signal model in use depends on the velocity of the target and the period of observations. If all the observations are collected in a short period and the velocity is small, then one can simply assume $g_{m}^{o}\left(t\right)\approx g_{m}^{o}\left(t_{0}\right)$ as [6]. Under the signal model (3), more observations can be used to make an estimation of the target space locations. If the observations are obtained in a long period and the velocity is huge, then this model may also mismatch and higher order approximations may be used.

For the *n*th angle-only passive sensors, the *l*th observation at $t_{k,n}$ is indexed by a triple $\left(l,k,n\right),l=1,\cdots ,L_{k,n}$. For simplicity, we also encode all the triples available, corresponding to all the observations available, with a one-to-one function Ω(l,k,n)→i. Then we define a set $L_{k,n}$ by

(4) $$L_{k,n}=\left\{i|i=\left(l,k,n\right),l=1,\cdots ,L_{k,n}\right\},k=1,\cdots ,N_{n},n=1,\cdots ,N$$

denotes a set of signal indices detected at the instant $t_{k,n}$ by the *n*th sensor. Therefore, $L_{k,n}=\left|L_{k,n}\right|$, where |·| over a set denotes the cardinality of the set. As the possibility of miss detection, false alarms and overlapping of signal sources, $L_{k,n}$ may not be equal to *M*. Denote

(5) $$L_{n}=\cup_{k=1}^{N_{n}}L_{k,n},L=\cup_{n=1}^{N}L_{n}$$

where ∪ denotes the union operation. The total number of observations by *N* sensors is denoted by

(6) $$N_{s}=\left|L\right|=\sum_{n=1}^{N}\sum_{k=1}^{N_{n}}L_{k,n}.$$

Each observation is associated with one of *M* targets or the false alarm indexed by 0, represented by a set M={0,1,⋯,M}. It can be considered as a mapping ψ:L→M, which is a correct mapping and is thus typically unknown in practice. According to our setting, the index set L can be partitioned into M+1 disjoint sets $A_{0},A_{1},\cdots ,A_{M}$, and $A_{m}$ is defined by

(7) $$A_{m}=\left\{i|\psi \left(i\right)=m,i\in L\right\},$$

where $A_{0}$ denotes the index of observations corresponding to false alarms, and $A_{m}$ denotes the index set of observations from the *m*th signal source. As a partition of A, we have $A_{i}\cap A_{j}=\emptyset$, i,j∈M, i≠j, and $A=\cup_{i=0}^{M}A_{i}$, where ∩ denotes the intersection operation of sets. Assume that $|A_{m}|=L_{m}$ and there are totally $L=\sum_{m=0}^{M}L_{m}$ observations available.

The signal indices in $A_{m}$ are composed of signal indices from all the sensors and the sub set for the *n*th sensor is denoted by

(8) $$A_{n,m}=L_{n}\cap A_{m}$$

which indicates the observations from the *n*th sensor probing the *m*th target. Denote $K_{n,m}=\left|A_{n,m}\right|$ and then we have $L_{m}=\sum_{n=1}^{N}K_{n,m}$ and $A_{m}=\cup A_{n,m}$.

For simplicity, we first assume that the mapping ψ is exactly known and then observations associated with $A_{m}$ is exactly known. For observation $i\in A_{m}$, real azimuth angle and elevation angle, regarding the *n*th sensor at $t_{k,n}$, can be expressed by

(9) $$\begin{array}{cc} \theta_{i}^{o} & =\tan^{-1}\left(y_{m,g}^{o}\left(t_{k,n}\right)-y_{n,s}^{o}\left(t_{k,n}\right),x_{m,g}^{o}\left(t_{k,n}\right)-x_{n,s}^{o}\left(t_{k,n}\right)\right)\\ \varphi_{i}^{o} & =\arctan\frac{z_{m,g}^{o}\left(t_{k,n}\right)-z_{n,s}^{o}\left(t_{k,n}\right)}{\sqrt{{\left(x_{m,g}^{o}\left(t_{k,n}\right)-x_{n,s}^{o}\left(t_{k,n}\right)\right)}^{2}+{\left(y_{m,g}^{o}\left(t_{k,n}\right)-y_{n,s}^{o}\left(t_{k,n}\right)\right)}^{2}}} \end{array}$$

respectively, where $\theta_{i}^{o}\in \left(-\pi ,\pi \right)$, $\varphi_{i}^{o}\in \left(-\frac{\pi }{2},\frac{\pi }{2}\right)$, $\tan^{-1}\left(*\right)$ is called the two-argument inverse tangent function [24,25] and arctan(∗) is the inverse tangent function. Denote $\eta_{i}^{o}={\left[\theta_{i}^{o},\varphi_{i}^{o}\right]}^{T}$. The azimuth angle and elevation angle measures can be written as

(10) $$\begin{array}{cc} \eta_{i} & ={\left[\theta_{i},\varphi_{i}\right]}^{T}=\eta_{i}^{o}+\Delta \eta_{i} \end{array}$$

(11) $$\begin{array}{cc} \theta_{i} & =\theta_{i}^{o}+\Delta \theta_{i} \end{array}$$

(12) $$\begin{array}{cc} \varphi_{i} & =\varphi_{i}^{o}+\Delta \varphi_{i} \end{array}$$

(13) $$\begin{array}{cc} \Delta \eta_{i} & ={\left[\Delta \theta_{i},\Delta \varphi_{i}\right]}^{T} \end{array}$$

where $\Delta \theta_{i}$ and $\Delta \varphi_{i}$ represent the measurement noise of the azimuth angle and elevation angle, respectively.

For simplicity, we assume that observation noises $\Delta \theta_{i}$ and $\Delta \varphi_{i}$ are statistically independent and follow zero-mean Gaussian distribution. The covariance matrices of Δη are denoted by $C_{\eta }\left(i\right)=E\left(\Delta \eta \Delta \eta^{T}\right)\in C^{2\times 2}$, namely $\Delta \eta ∼N(0,C_{\eta }\left(i\right))$, which is typically affected by the SNR of the signal, where $N(0,C_{\eta }\left(i\right))$ denotes the zero-mean Gaussian distribution with mean 0 and covariance matrix $C_{\eta }\left(i\right)$.

### 2.2. Estimation of Target Track

Each angle-only observation contributes a line in 3D space and without measurement error, a target will be present at the line. With many angle-only observations, real position of the target can be determined. The line associated with the *i*th observation can be expressed by

(14) $$\begin{array}{c} L_{i}:x\left(t_{i}\right)=p_{i}+\alpha_{i}e_{i},\alpha_{i}\in R \end{array}$$

where $p_{i}$ denotes the sensor location regarding the *i*th observation, $\alpha_{i}$ is a parameter indicating the distance to the origin $p_{i}$, $e_{i}=e\left(\eta_{i}\right)={\left[e_{i,x},e_{i,y},e_{i,z}\right]}^{T}\in R^{3\times 1}$ is the normalized direction vector associated with the angle observation $\eta_{i}$, namely $∥e_{i}∥=1$, ∥·∥ over a vector denotes the $\ell_{2}$-norm, and

(15) $$\begin{array}{cc} e_{i,x} & =\cos\left(\theta_{i}\right)\cos\left(\varphi_{i}\right) \end{array}$$

(16) $$\begin{array}{cc} e_{i,y} & =\sin\left(\theta_{i}\right)\cos\left(\varphi_{i}\right) \end{array}$$

(17) $$\begin{array}{cc} e_{i,y} & =\sin(\varphi_{i}). \end{array}$$

In what follows, we consider the observations in $A_{m},m\neq 0$. From (3), we can rewrite

(18) $$x\left(t_{i}\right)=p_{i}+\alpha_{i}e_{i}=g_{0,m}+v_{g,m}\left(t_{i}-t_{0}\right)+\epsilon_{e}\left(i\right)=A_{e}\left(i\right)q_{m}+\epsilon_{e}\left(i\right)$$

where $q_{m}={\left[g_{0,m}^{T},v_{g,m}^{T}\right]}^{T},g_{0,m}=g_{m}^{o}\left(t_{0}\right),v_{g,m}=v_{m}^{o}\left(t_{0}\right)$, $\epsilon_{e}\left(i\right)$ denotes the bias term,

(19) $$A_{e}\left(i\right)=\left[I,\left(t_{i}-t_{0}\right)I\right]\in R^{3\times 6}$$

and I denotes the identity matrix. In (18), there are totally 7 unknown parameters and an observation can provide 3 equations. In addition to an observation, one can obtain another 3 equations and the number of unknown parameters will increase by 1. Unless specified, we always refer to the *m*th target and drop the subscripts *m* in situations without ambiguity subsequently, e.g., denote $g_{0,m}\to g_{0},v_{g,m}\to v_{g},q_{m}\to q$.

In order to determine the location and velocity of the target, the optimization problem can be formulated as

(20) $$\min_{\alpha ,q}∥P+E\mathrm{diag}\left(\alpha \right)-A_{h}\left(1_{6}⊗q\right)∥$$

where ∥·∥ refers to the $\ell_{2}—$norm subsequently unless explicitly specified, $\alpha ={\left[\alpha_{1},\cdots ,\alpha_{L_{m}}\right]}^{T}$, P is a matrix whose columns are $p_{i},i\in A_{m}$, $P=[p_{,}\cdots ,p_{L_{m}}]$, $1_{6}$ denotes a 6×1 all-one vector of length $L_{m}$, E is a matrix whose columns are $e_{i},i\in A_{m}$, ⊗ denotes the Kronecker product operation,

(21) $$A_{h}=\left[A_{e}\left(i\right),\cdots \right]\in R^{3\times 6L_{m}},i\in A_{m},$$

and diag(·) with a vector entry denotes a diagonal matrix with the vector as diagonal elements.

### 2.3. The Gross LS Algorithm

In practice, the observations are generally contaminated by measurement noise and then the lines often do not intersect into a point in space. With $L_{m}$ observations, there are totally $L_{m}+6$ unknown parameters and $3L_{m}$ equations. Therefore, if we have at least 3 observations from angle-only sensors, we can find 9 unknown parameters together. For that purpose, let $\bar{q}={\left[q^{T},\alpha^{T}\right]}^{T}$ and then we can reformulate (18). In order to minimize the mismatch, the optimization problem can be rewritten as

(22) $$\min_{\alpha ,q}∥p+D\alpha -A_{d}q∥.$$

The combination of equations for all observation indices in $A_{m}$ can be formulated as

(23) $$A_{a}\bar{q}=p+\epsilon_{e}$$

where p=vec(P), vec(·) denotes the vectorization operation.

(24) $$\begin{array}{cc} \epsilon_{e} & ={\left[\epsilon^{T}\left(1\right),\cdots ,\epsilon^{T}\left(L_{m}\right)\right]}^{T} \end{array}$$

(25) $$\begin{array}{cc} A_{a} & =\left[A_{d},-D\right]\in R^{3L_{m}\times \left(6+L_{m}\right)} \end{array}$$

(26) $$\begin{array}{cc} A_{d} & ={\left[A_{e}^{T}\left(1\right),\cdots ,A_{e}^{T}\left(L_{m}\right)\right]}^{T} \end{array}$$

(27) $$\begin{array}{cc} \; & =\left[1,t\right]⊗I\in R^{3L_{m}\times 3} \end{array}$$

(28) $$\begin{array}{cc} D & =\mathrm{diag}\left(e_{1},\cdots ,e_{L_{m}}\right)\in R^{3L_{m}\times L_{m}} \end{array}$$

where $t={\left[t_{1}-t_{0},t_{2}-t_{0},\ldots ,t_{L_{m}}-t_{0}\right]}^{T}$, and diag(·) with some matrices inputs denotes a block diagonal matrix with the input matrices as block diagonal elements.

For this optimization problem, we can find the classical LS solution as

(29) $${\hat{q}}_{a}={\left(A_{a}^{T}A_{a}\right)}^{-1}A_{a}^{T}p.$$

It can be proved that for symmetric matrices A and B, and C, all of appropriate sizes,

(30) $${\left[\begin{array}{cc} A & C^{T}\\ C & B \end{array}\right]}^{-1}=\left[\begin{array}{cc} {\left(A-C^{T}B^{-1}C\right)}^{-1} & -{\left(A-C^{T}B^{-1}C\right)}^{-1}C^{T}B\\ -B^{-1}C{\left(A-C^{T}B^{-1}C\right)}^{-1} & {\left(B-CA^{-1}C^{T}\right)}^{-1} \end{array}\right]$$

Consequently, with a fact that $D^{T}D=I$, we have

(31) $${\left(A_{a}^{T}A_{a}\right)}^{-1}={\left[\begin{array}{cc} A_{d}^{T}A_{d} & -A_{d}^{T}D\\ -D^{T}A_{d} & D^{T}D \end{array}\right]}^{-1}=\left[\begin{array}{cc} X & Z^{T}\\ Z & Y \end{array}\right]$$

where

(32) $$\begin{array}{cc} X & ={\left(A_{d}^{T}A_{d}-A_{d}^{T}D{\left(D^{T}D\right)}^{-1}D^{T}A_{d}\right)}^{-1} \end{array}$$

(33) $$\begin{array}{cc} \; & ={\left(A_{d}^{T}A_{d}-A_{d}^{T}DD^{T}A_{d}\right)}^{-1}\in R^{6\times 6} \end{array}$$

(34) $$\begin{array}{cc} Y & ={\left(D^{T}D-D^{T}A_{d}{\left(A_{d}^{T}A_{d}\right)}^{-1}A_{d}^{T}D\right)}^{-1} \end{array}$$

(35) $$\begin{array}{cc} \; & ={\left(I-D^{T}A_{d}{\left(A_{d}^{T}A_{d}\right)}^{-1}A_{d}^{T}D\right)}^{-1}\in R^{L_{m}\times L_{m}} \end{array}$$

(36) $$\begin{array}{cc} Z & ={\left(D^{T}D\right)}^{-1}D^{T}A_{d}X \end{array}$$

(37) $$\begin{array}{cc} \; & =D^{T}A_{d}{\left(A_{d}^{T}A_{d}-A_{d}^{T}DD^{T}A_{d}\right)}^{-1}\in R^{L_{m}\times 3} \end{array}$$

and the following equation is used in above formulations.

It can also be proved that

(38) $$\begin{array}{cc} A_{d}^{T}A_{d} & =\left[\begin{array}{cc} LI & T_{d}I\\ T_{d}I & T_{D}I \end{array}\right]=M⊗I \end{array}$$

(39) $$\begin{array}{cc} A_{d}^{T}D & =\left[\begin{array}{c} E\\ E_{t} \end{array}\right], \end{array}$$

where $T_{d}=\sum_{i=1}^{L}\left(t_{i}-t_{0}\right),T_{D}=\sum_{i=1}^{L}{\left(t_{i}-t_{0}\right)}^{2}$, $E_{t}=E\mathrm{diag}\left(t\right)$

(40) $$M=\left[\begin{array}{cc} L & T_{d}\\ T_{d} & T_{D} \end{array}\right]\Leftrightarrow M^{-1}=\frac{1}{LT_{D}-T_{d}^{2}}\left[\begin{array}{cc} T_{D} & -T_{d}\\ -T_{d} & L \end{array}\right]$$

and ⊗ denotes the Kronecker product operation.

Still evoke (30) and we have

(41) $${\left(A_{d}^{T}A_{d}\right)}^{-1}=M^{-1}⊗I$$

and thus

(42) $$\begin{array}{cc} Y^{-1} & =I-\frac{1}{LT_{D}-T_{d}^{2}}\left[E^{T},E_{t}^{T}\right]\left[\begin{array}{c} T_{D}E-T_{d}E_{t}\\ -T_{d}E+LE_{t} \end{array}\right] \end{array}$$

(43) $$\begin{array}{cc} \; & =I-\frac{1}{LT_{D}-T_{d}^{2}}\left(T_{D}E^{T}E-T_{d}E_{t}^{T}E-T_{d}E_{t}^{T}E+LE_{t}^{T}E_{t}\right). \end{array}$$

Meanwhile,

(44) $$A_{a}^{T}p=\left[\begin{array}{c} A_{d}^{T}p\\ -D^{T}p \end{array}\right]=\left[\begin{array}{c} q_{t}\\ -p_{e} \end{array}\right]$$

where

(45) $$\begin{array}{cc} q_{t} & =P{\left[1,t^{T}\right]}^{T} \end{array}$$

(46) $$\begin{array}{cc} p_{e} & =\mathrm{diag}(E^{T}P). \end{array}$$

Therefore, the estimates for $q_{m}$ and α can be written separately as

(47) $$\begin{array}{cc} {\hat{q}}_{m} & =\left[\begin{array}{c} {\hat{g}}_{0}\\ {\hat{v}}_{g} \end{array}\right]=Xq_{t}-X\left[\begin{array}{c} E\\ E_{t} \end{array}\right]p_{e} \end{array}$$

(48) $$\begin{array}{cc} {\hat{\alpha }}_{m} & =A_{d}^{T}DXq_{t}-Yp_{e} \end{array}$$

where X can be written in a concise form as

(49) $$X^{-1}=\left[\begin{array}{cc} LI-EE^{T} & T_{d}I-EE_{t}^{T}\\ T_{d}I-E_{t}E^{T} & T_{D}I-E_{t}E_{t}^{T} \end{array}\right].$$

Under the assumption that all the sample under consideration is from the same target, the track, parameterized by q, is identical for all observations hereafter.

### 2.4. The Linear LS Algorithm

In a long period, one can obtain a sequence of η=(θ,ϕ) observations. If we take all the observations into consideration for optimization, a huge computation cost may be required. In some cases, it is also unnecessary at all. We can extract information from local observations and then transmit estimated parameters to the fusion center for target location estimation. The target location has been approximated by a linear model. Next, we express the DOA and sensor location by a linear model as well.

For DOA measures from a sensor, we can approximate a series of angle measures by

(50) $$\eta =\left\{\begin{array}{c} \theta \approx \theta_{0}+{\dot{\theta }}_{0}\left(t-t_{0}\right)\\ \varphi \approx \varphi_{0}+{\dot{\varphi }}_{0}\left(t-t_{0}\right). \end{array}\right.$$

Now consider the *n*th sensor and let

(51) $$\left\{\begin{array}{c} \theta_{0}\to \theta_{0,n}\\ {\dot{\theta }}_{0}\to {\dot{\theta }}_{0,n} \end{array}\right.\Rightarrow {\bar{\theta }}_{n}=\left[\begin{array}{c} \theta_{0,n}\\ {\dot{\theta }}_{0,n} \end{array}\right],\;\left\{\begin{array}{c} \varphi_{0}\to \varphi_{0,n}\\ {\dot{\varphi }}_{0}\to {\dot{\varphi }}_{0,n} \end{array}\right.\Rightarrow {\bar{\varphi }}_{n}=\left[\begin{array}{c} \varphi_{0,n}\\ {\dot{\varphi }}_{0,n} \end{array}\right].$$

For all observations in $A_{n,m}$, we can write a polynomial regression problem as

(52) $$\left\{\begin{array}{c} \theta_{n}=A_{n}{\bar{\theta }}_{n}+\epsilon_{\theta }\\ \varphi_{n}=A_{n}{\bar{\varphi }}_{n}+\epsilon_{\varphi } \end{array}\right.$$

where $\theta_{n}={\left[\theta_{i}\right]}_{i\in A_{n,m}},\varphi_{n}={\left[\varphi_{i}\right]}_{i\in A_{n,m}}$, $\epsilon_{\theta }$ denotes bias for $\theta_{n}$, $\epsilon_{\phi }$ denotes bias for $\varphi_{n}$, and

(53) $$A_{a}\left(n\right)=\left[\begin{array}{c} 1,t_{i}-t_{0}\\ \vdots \end{array}\right]\in R^{K_{n,m}\times 2},i\in A_{n,m}.$$

It can be proved that the LS estimate of the directions for the *n*th sensor can be directly written as

(54) $$\begin{array}{cc} {\hat{\theta }}_{n} & ={\left(A_{a}{\left(n\right)}^{T}A_{a}\left(n\right)\right)}^{-1}A_{a}{\left(n\right)}^{T}\theta_{n}=\frac{1}{LT_{D}-T_{d}^{2}}\left[\begin{array}{c} T_{D}\theta_{n}^{T}1-T_{d}\theta_{n}^{T}t\\ -T_{d}\theta_{n}^{T}1+L\theta_{n}^{T}t \end{array}\right] \end{array}$$

(55) $$\begin{array}{cc} {\hat{\varphi }}_{n} & ={\left(A_{a}{\left(n\right)}^{T}A_{a}\left(n\right)\right)}^{-1}A_{a}{\left(n\right)}^{T}\varphi_{n}=\frac{1}{LT_{D}-T_{d}^{2}}\left[\begin{array}{c} T_{D}\varphi_{n}^{T}1-T_{d}\varphi_{n}^{T}t\\ -T_{d}\varphi_{n}^{T}1+L\varphi_{n}^{T}t \end{array}\right]. \end{array}$$

In this case, ${\hat{\theta }}_{n}$ and ${\hat{\varphi }}_{n}$, instead of $\theta_{n}$ and $\varphi_{n}$, will be transmitted to the fusion center, such that the communication cost will be greatly reduced.

In (18), ${\hat{\theta }}_{n}$ and ${\hat{\varphi }}_{n}$ affects the positioning accuracy through the normalized direction vector $e_{i},i\in A_{n,m}$. With a linear approximation model, e can be rewritten as

(56) $$e\left(t\right)\approx e_{0}+{\dot{E}}_{0}^{T}{\dot{\eta }}_{0}\left(t-t_{0}\right)$$

where $e_{0}=e\left(t_{0}\right)$,

(57) $$\begin{array}{cc} \dot{\theta } & =\frac{\partial \theta }{\partial t},\dot{\varphi }=\frac{\partial \varphi }{\partial t} \end{array}$$

(58) $$\begin{array}{cc} \eta_{0} & ={\left[\theta_{0},\varphi_{0}\right]}^{T},\eta ={\left[\theta ,\varphi \right]}^{T} \end{array}$$

(59) $$\begin{array}{cc} {\dot{\eta }}_{0} & =\dot{\eta }{|}_{t=t_{0}}={\left[{\dot{\theta }}_{0},{\dot{\varphi }}_{0}\right]}^{T},\dot{\eta }={\left[\dot{\theta },\dot{\varphi }\right]}^{T} \end{array}$$

(60) $$\begin{array}{cc} {\dot{E}}_{0} & =\dot{E}{\left(\eta \right)|}_{t=t_{0}}=\dot{E}\left(\eta_{0}\right) \end{array}$$

and $\dot{E}\left(\eta \right)$ denotes the Jacobi matrix defined by

(61) $${\dot{E}}^{T}\left(\eta \right)=\frac{\partial e}{\partial \eta^{T}}=\left[\begin{array}{cc} -\sin(\theta )\cos(\varphi ) & -\cos(\theta )\sin(\varphi )\\ \cos(\theta )\cos(\varphi ) & -\sin(\theta )\sin(\varphi )\\ 0 & \cos(\varphi ). \end{array}\right]$$

For the *n*th sensor, denote $e_{0}\to e_{0,n},{\dot{E}}_{0}\to {\dot{E}}_{0,n}$, ${\dot{\eta }}_{0}\to {\dot{\eta }}_{0,n}$, $\eta_{0}\to \eta_{0,n}$ and so on.

In practice, the position of the platform is also measured by a device, such as an inertial system or a positioning system. In either case, if the *n*th sensor is moving with speed $v_{s}$ at $t=t_{0}$, the position can be expressed by

(62) $$p_{n}\left(t\right)=p_{0,n}+v_{s,n}\left(t-t_{0}\right)+\epsilon_{s}\left(n\right)$$

where $p_{0,n}$ denotes the position of the *n*th sensor, $v_{s,n}$ denotes the velocity, both at $t=t_{0}$, and $\epsilon_{s}$ denotes the bias of position estimation error.

For simplicity, we assume that the sensor location is measured at $t={\bar{t}}_{k,n},k=1,\cdots ,{\bar{N}}_{n}$. In practice, in this configuration, the sensor location can be measured at instants other than $t_{k,n},k=1,\cdots ,N_{n}$. Now we can construct equations as

(63) $$p_{k,n}=p_{0,n}+v_{s,n}\left({\bar{t}}_{k,n}-t_{0}\right)+\epsilon_{s}\left(k,n\right),k=1,\cdots ,{\bar{N}}_{n},n=1,\cdots ,N$$

or in another form as

(64) $$p_{s,n}=A_{s}{\bar{p}}_{n}+\epsilon_{s}\left(n\right)$$

where ${\bar{p}}_{n}={\left[p_{0,n}^{T},v_{s,n}^{T}\right]}^{T}$, $p_{s,n}={\left[p_{1,n}^{T},\cdots ,p_{{\bar{N}}_{n},n}^{T}\right]}^{T}$, and

(65) $$A_{s}\left(n\right)={\left[\begin{array}{c} I,({\bar{t}}_{k,n}-t_{0})I\\ \vdots \end{array}\right]}_{k=1,\cdots ,{\bar{N}}_{n}}=\left[1,t_{s}\right]⊗I$$

for which

(66) $${\left(A_{s}^{T}\left(n\right)A_{s}\left(n\right)\right)}^{-1}=M_{s}^{-1}⊗I$$

where $M_{s}^{-1}$ is similar to M in (40).

The LS estimate of $\bar{p}$ is

(67) $$\begin{array}{cc} {\hat{p}}_{n} & =\left[\begin{array}{c} {\hat{p}}_{0,n}^{T}\\ {\hat{v}}_{s,n} \end{array}\right]={\left(A_{s}^{T}\left(n\right)A_{s}\left(n\right)\right)}^{-1}A_{s}^{T}\left(n\right)p_{s,n} \end{array}$$

(68) $$\begin{array}{cc} \; & =\left(M_{s}^{-1}⊗I\right)\left[\begin{array}{c} P1\\ P_{t}t \end{array}\right] \end{array}$$

(69) $$\begin{array}{cc} {\hat{p}}_{0,n} & =\frac{1}{LT_{D}-T_{d}^{2}}\left(T_{D}P1-T_{d}P_{t}t\right) \end{array}$$

and

(70) $$\begin{array}{cc} {\hat{v}}_{s,n} & =\frac{1}{LT_{D}-T_{d}^{2}}\left(-T_{d}P1+LP_{t}t\right). \end{array}$$

With above operations, we can obtain a linear sensor location parameter ${\hat{p}}_{n}$, linear DOA parameters ${\hat{\theta }}_{n},{\hat{\phi }}_{n}$, and linear target location parameters $q_{m}$. The accuracy depends on the interval of observations, the speed of the target, and the speed of the sensors. A series of observations can now be approximated by two parameters and it is now unnecessary to transmit all the local observation to a fusion center anymore.

With only one observation available, a fusion center can now estimate the target position and velocity with the following equation

(71) $$\begin{array}{cc} x(t) & =g_{0}+v_{g}\left(t-t_{0}\right)+\epsilon_{a}\left(n\right)=p_{0,n}+v_{s,n}\left(t-t_{0}\right)+\alpha_{n}\left(t\right)e_{n}\left(t\right)\\ \; & \approx p_{0,n}+v_{s,n}\left(t-t_{0}\right)+\left(\alpha_{0,n}+{\dot{\alpha }}_{0,n}\left(t-t_{0}\right)\right)\left(e_{0,n}+{\dot{E}}_{0,n}^{T}{\dot{\eta }}_{0,n}\left(t-t_{0}\right)\right) \end{array}$$

where ${\dot{\alpha }}_{0,n}$ represents the change rate of $\alpha_{0,n}$. By expanding Equation (71) and ignoring the second-order term, $\epsilon_{a}\left(n\right)$ can be reformulated as

(72) $$\begin{array}{cc} \epsilon_{a}\left(n\right) & =p_{0,n}+\alpha_{0,n}e_{0,n}-g_{0}+\left(\alpha_{0,n}{\dot{E}}_{0,n}^{T}{\dot{\eta }}_{0,n}+v_{s,n}+{\dot{\alpha }}_{0,n}e_{0,n}-v_{g}\right)\left(t-t_{0}\right) \end{array}$$

where n=1,2…,N and $\epsilon_{a}\left(n\right)$ denotes the bias term in approximation target position and direction of arrival by the linear models.

The following equation can be obtained using (72) for *N* sensors,

(73) $$\begin{array}{c} \epsilon_{a}=p_{0}+D_{0}\alpha_{0}-\bar{I}g_{0}+\left(X_{d}\alpha_{0}+v_{s}+D_{0}{\dot{\alpha }}_{0}-\bar{I}v_{g}\right)\left(t-t_{0}\right) \end{array}$$

where

(74) $$\begin{array}{cc} \alpha_{0} & ={\left[\alpha_{0,1},\alpha_{0,2},\cdots ,\alpha_{0,N}\right]}^{T} \end{array}$$

(75) $$\begin{array}{cc} {\dot{\alpha }}_{0} & ={\left[{\dot{\alpha }}_{0,1},{\dot{\alpha }}_{0,2},\cdots ,{\dot{\alpha }}_{0,N}\right]}^{T} \end{array}$$

(76) $$\begin{array}{cc} p_{0} & ={\left[p_{0,1}^{T},p_{0,2}^{T},\ldots ,p_{0,N}^{T}\right]}^{T} \end{array}$$

(77) $$\begin{array}{cc} D_{0} & =\mathrm{diag}(e_{0,1},e_{0,2},\ldots ,e_{0,N}) \end{array}$$

(78) $$\begin{array}{cc} X_{d} & =\mathrm{diag}({\dot{E}}_{0,1}^{T}{\dot{\eta }}_{0,1},{\dot{E}}_{0,2}^{T}{\dot{\eta }}_{0,2},\ldots ,{\dot{E}}_{0,N}^{T}{\dot{\eta }}_{0,N}) \end{array}$$

(79) $$\begin{array}{cc} v_{s} & ={\left[v_{s,1}^{T},v_{s,2}^{T},\ldots ,v_{s,N}^{T}\right]}^{T} \end{array}$$

(80) $$\begin{array}{cc} \bar{I} & =1_{N}⊗I_{3}\in R^{3N\times 3} \end{array}$$

and

(81) $$\begin{array}{cc} \epsilon_{a} & ={\left[\epsilon_{a}^{T}\left(1\right),\epsilon_{a}^{T}\left(2\right),\cdots ,\epsilon_{a}^{T}\left(N\right)\right]}^{T}. \end{array}$$

In order to minimize the total bias $\epsilon_{a}$, we can minimize

(82) $$\begin{array}{c} \min_{q_{c},q_{v}}∥p_{0}+D_{0}\alpha_{0}-\bar{I}g_{0}+\left(X_{d}\alpha_{0}+v_{s}+D_{0}{\dot{\alpha }}_{0}-\bar{I}v_{g}\right)\left(t-t_{0}\right)∥,t\in T. \end{array}$$

where $q_{c}={\left[g_{0}^{T},\alpha_{0}^{T}\right]}^{T}$ and $q_{v}={\left[v_{g}^{T},{\dot{\alpha }}_{0}^{T}\right]}^{T}$.

To ensure the bias is minimized for t∈T, both the initial position bias, $p_{0}+D_{0}\alpha_{0}-\bar{I}g_{0}$, and the speed bias, $X_{d}\alpha_{0}+v_{s}+D_{0}{\dot{\alpha }}_{0}-\bar{I}v_{g}$ should be minimized. Therefore, we can solve the optimization problem through solving the following two optimization problems of smaller scale,

(83) $$\begin{array}{cc} \; & \min_{q_{c}}∥p_{0}+D_{0}\alpha_{0}-\bar{I}g_{0}∥ \end{array}$$

(84) $$\begin{array}{cc} \; & \min_{q_{v}}∥X_{d}\alpha_{0}+v_{s}+D_{0}{\dot{\alpha }}_{0}-\bar{I}v_{g}∥. \end{array}$$

The solutions to the problems can be found directly through the LS algorithm as

(85) $$\begin{array}{cc} {\hat{q}}_{c} & ={\left[{\hat{g}}_{0}^{T},{\hat{\alpha }}_{0}^{T}\right]}^{T}={\left(A_{c}^{T}A_{c}\right)}^{-1}A_{c}^{T}p_{0} \end{array}$$

(86) $$\begin{array}{cc} {\hat{q}}_{v} & ={\left[{\hat{v}}_{g}^{T},{\hat{\dot{\alpha }}}_{0}^{T}\right]}^{T}={\left(A_{c}^{T}A_{c}\right)}^{-1}A_{c}^{T}\left(X_{d}\alpha_{0}+v_{s}\right) \end{array}$$

where

(87) $$\begin{array}{cc} A_{c} & =[1_{N}⊗I_{3},-D_{0}]. \end{array}$$

It can be proved that

(88) $$\begin{array}{c} A_{c}^{T}A_{c}=\left[\begin{array}{cc} {\bar{I}}^{T}\bar{I} & -{\bar{I}}^{T}D_{0}\\ -D_{0}^{T}\bar{I} & D_{0}^{T}D_{0} \end{array}\right]=\left[\begin{array}{cc} NI_{3} & -E_{0}\\ -E_{0}^{T} & I \end{array}\right] \end{array}$$

and then

(89) $${\left(A_{c}^{T}A_{c}\right)}^{-1}=\left[\begin{array}{cc} {\left(NI-E_{0}E_{0}^{T}\right)}^{-1} & {\left(NI-E_{0}E_{0}^{T}\right)}^{-1}E_{0}\\ E_{0}^{T}{\left(NI-E_{0}E_{0}^{T}\right)}^{-1} & I+E_{0}^{T}{\left(NI-E_{0}E_{0}^{T}\right)}^{-1}E_{0} \end{array}\right].$$

where $E_{0}=\left[e_{0,1},e_{0,2},\ldots ,e_{0,N}\right]$ and $P_{0}=\left[p_{0,1},p_{0,2},\ldots ,p_{0,N}\right]$.

Meanwhile,

(90) $$\begin{array}{c} A_{c}^{T}p_{0}=\left[\begin{array}{c} P_{0}1\\ -D_{0}^{T}p_{0} \end{array}\right] \end{array}$$

and thus,

(91) $$\begin{array}{cc} {\hat{q}}_{0} & ={\left[{\hat{g}}_{0}^{T},{\hat{\alpha }}_{0}^{T}\right]}^{T}={\left(A_{c}^{T}A_{c}\right)}^{-1}A_{c}^{T}p_{0}\\ {\hat{g}}_{0} & ={\left(NI-E_{0}E_{0}^{T}\right)}^{-1}P_{0}1-{\left(NI-E_{0}E_{0}^{T}\right)}^{-1}E_{0}D_{0}^{T}p_{0} \end{array}$$

(92) $$\begin{array}{cc} \; & ={\left(NI-E_{0}E_{0}^{T}\right)}^{-1}\left(P_{0}1-E_{0}D_{0}^{T}p_{0}\right)\\ {\hat{\alpha }}_{0} & =D_{0}^{T}\bar{I}{\hat{g}}_{0}-D_{0}^{T}p_{0} \end{array}$$

(93) $$\begin{array}{cc} \; & =D_{0}^{T}\bar{I}{\left(NI-E_{0}E_{0}^{T}\right)}^{-1}\left(P_{0}1-E_{0}D_{0}^{T}p_{0}\right)-D_{0}^{T}p_{0} \end{array}$$

which is identical to the solution of (82). A minor difference is that the matrix inverse operation is over an N×N matrix $NI-E_{0}E_{0}^{T}$.

The solution to $q_{v}$ can be expressed by

(94) $$\begin{array}{cc} A_{c}^{T}\left(X_{d}\alpha_{0}+v_{s}\right) & =\left[\begin{array}{c} 1_{N}^{T}⊗I_{3}\\ -D_{0}^{T} \end{array}\right]\left(\mathrm{vec}\left(X\mathrm{diag}\left(\alpha_{0}\right)\right)+\mathrm{vec}\left(V_{s}\right)\right)\\ \; & =\left[\begin{array}{c} X\alpha_{0}+V_{s}1\\ -v_{a}-v_{e} \end{array}\right] \end{array}$$

where

(95) $$\begin{array}{cc} X & =[{\dot{E}}_{0,1}^{T}{\dot{\eta }}_{0,1}{\dot{E}}_{0,2}^{T}{\dot{\eta }}_{0,2},\ldots ,{\dot{E}}_{0,N}^{T}{\dot{\eta }}_{0,N}] \end{array}$$

(96) $$\begin{array}{cc} V_{s} & =[v_{s,1},v_{s,2},\ldots ,v_{s,N}]\\ v_{a} & =\mathrm{diag}\left(\alpha_{0}\right)E_{0}^{T}X \end{array}$$

(97) $$\begin{array}{cc} \; & ={\left[\alpha_{0,1}e_{0,1}^{T}{\dot{E}}_{0,1}^{T}{\dot{\eta }}_{0,1},\cdots ,\alpha_{0,N}e_{0,N}^{T}{\dot{E}}_{0,N}^{T}{\dot{\eta }}_{0,N}\right]}^{T} \end{array}$$

and

(98) $$\begin{array}{cc} v_{e} & ={\left[e_{0,1}^{T}v_{s,1},\cdots ,e_{0,N}^{T}v_{s,N}\right]}^{T}. \end{array}$$

Consequently, we can obtain

(99) $$\begin{array}{cc} {\hat{v}}_{g} & ={\left(NI-E_{0}E_{0}^{T}\right)}^{-1}\left(X\alpha_{0}+V_{s}1-E_{0}v_{a}-E_{0}v_{e}\right) \end{array}$$

(100) $$\begin{array}{cc} {\hat{\dot{\alpha }}}_{0} & =E_{0}^{T}{\left(NI-E_{0}E_{0}^{T}\right)}^{-1}\left(X\alpha_{0}+V_{s}1\right)-v_{a}-v_{e}-E_{0}^{T}{\left(NI-E_{0}E_{0}^{T}\right)}^{-1}E_{0}\left(v_{a}+v_{e}\right) \end{array}$$

(101) $$\begin{array}{cc} \; & =E_{0}^{T}{\left(NI-E_{0}E_{0}^{T}\right)}^{-1}\left(X\alpha_{0}+V_{s}1-E_{0}v_{a}-E_{0}v_{e}\right)-v_{a}-v_{e} \end{array}$$

(102) $$\begin{array}{cc} \; & =E_{0}^{T}{\hat{v}}_{g}-v_{a}-v_{e}. \end{array}$$

One can also derive in another way. According to (93), the change rate ${\dot{\alpha }}_{0}$ of $\alpha_{0}$ can be expressed as

(103) $$\begin{array}{c} {\dot{\alpha }}_{0}=X_{d}^{T}\bar{I}{\hat{g}}_{0}+D_{0}^{T}\bar{I}v_{g}-X_{d}^{T}p_{0}-D_{0}^{T}v_{s}. \end{array}$$

Take (103) into (84), which can be rewritten as

(104) $$\begin{array}{c} \min_{v_{g}}∥X_{d}{\hat{\alpha }}_{0}+v_{s}+D_{0}X_{d}^{T}\bar{I}{\hat{g}}_{0}-D_{0}D_{0}^{T}v_{s}-D_{0}X_{d}^{T}p_{0}+\left(D_{0}D_{0}^{T}-I\right)\bar{I}v_{g}∥. \end{array}$$

The solution of (104) can also be found through the LS algorithm, which can be expressed as

(105) $$\begin{array}{c} {\hat{v}}_{g}={\left(A_{v}^{T}A_{v}\right)}^{-1}A_{v}^{T}b_{v} \end{array}$$

where

(106) $$\begin{array}{cc} A_{v} & =\left(I-D_{0}D_{0}^{T}\right)\bar{I} \end{array}$$

(107) $$\begin{array}{cc} b_{v} & =X_{d}{\hat{\alpha }}_{0}+v_{s}+D_{0}X_{d}^{T}\bar{I}{\hat{g}}_{0}-D_{0}D_{0}^{T}v_{s}-D_{0}X_{d}^{T}p_{0}. \end{array}$$

### 2.5. The Truncated LS Algorithm

For a better performance, it is necessary to estimate the change rate $\dot{\alpha }$ and if we ignore this term, the optimization problem becomes

(108) $$\min_{v_{g}}∥X_{d}\alpha_{0}+v_{s}-\bar{I}v_{g}∥$$

whose solution, termed as truncated LS algorithm subsequently, is

(109) $${\hat{v}}_{g}=\frac{1}{N}\left(V_{s}1+X{\hat{\alpha }}_{0}\right)$$

which is an average operation. Note that the truncated LS algorithm shares the same position estimate with the linear LS algorithm.

The estimate of the target location at t∈T can be written as

(110) $${\hat{g}}_{m}\left(t\right)={\hat{g}}_{0}+{\hat{v}}_{g}\left(t-t_{0}\right),t\in T.$$

In (99) and (109), the velocity terms $v_{s,k},k=1,\cdots ,N$ are unknown and should be replaced by their estimates, typically ${\hat{v}}_{s}$ estimated in the LS algorithm as in (70). In practice, besides the linear regression method performed at local sensors, there may be other methods that can output more accurate velocity and angle difference information. For instance, some inertial devices can measure the velocity more accurately than the LS algorithm in use. With a more accurate velocity estimate, it is possible to obtain a better positioning performance.

Both the linear LS algorithm and the truncated LS algorithm estimate the velocity of the target and thus can make the time-consuming nonlinear filtering operation update in a longer time interval. Subsequently, the performances of these algorithms will be analyzed in numerical results.

## 3. Numerical Results

**Figure 2:**
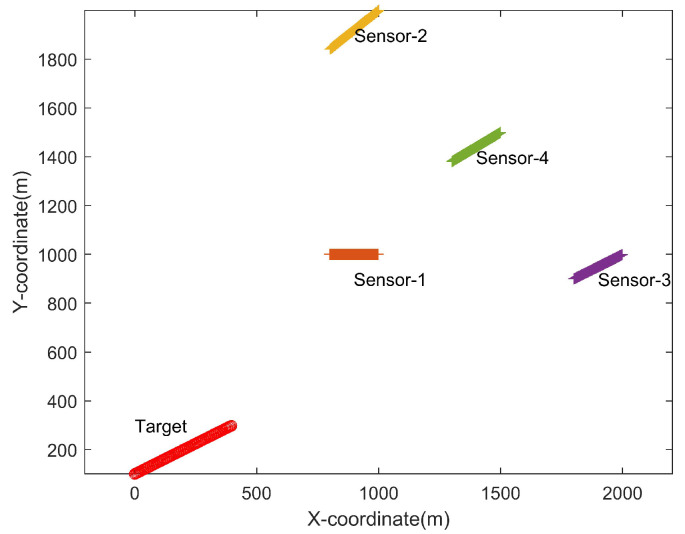
The topology of the sensors and the target.

In order to evaluate the performance of the concerned positioning algorithms, we first consider a scenario where four angle-only sensors are estimating the position of a target with their angle-only observations. Both the sensors and the target are moving with a constant speed during the period of observation by assumption. The initial position and the constant speed of the sensors and the target are shown in Table 1. The scenario is illustrated in Figure 2.

All the sensors output observations at a frequency of 50 Hz, i.e., with a period of 20 ms. But they operate on an asynchronous manner, namely the sensors record the observations at independent instants. The differences of the sampling instants are randomly generated within 20ms. This assumption is important in real situations because it allows distributed sensors to operate asynchronously. We also assume that there is no error in recording the instants of the observations and for all the sensors, no signal is missed in detection during the observation period.

Assume that the self-positioning error is distributed with zero-mean normal distribution, whose variance is 1m for all the sensors, namely

(111) $$C_{s}\left(k,n\right)=E\left(\Delta p_{n}\left(t_{k,n}\right)\Delta p_{n}^{T}\left(t_{k,n}\right)\right)=I,n=1,\cdots ,N.$$

The angle measurement error also follows zero-mean normal distribution with variance of 0.5 degree for all the observations, namely

(112) $$C_{\eta }\left(i\right)=E\left(\Delta \eta_{i}\Delta \eta_{i}^{T}\right)=0.5I,i\in A_{m}.$$

At the current stage, we do not consider the measurement errors from the gyroscopes installed on the platforms along with the sensors. Therefore, the angle measurement error is caused by the sensors only.

In order to evaluate the performance of the algorithms, we run $N_{e}=20$ random experiments and take the root mean square error (RMSE) as the resulting performance metric. The RMSE of position, RMSE of velocity and the gross RMSE at instant *t* in scale and in dB are defined by

(113) $$\begin{array}{ccc} {\mathrm{RMSE}}_{p}\left(t\right) & =\sqrt{\frac{1}{N_{e}}\sum_{k=1}^{N_{e}}{\left|{\hat{g}}_{0}\left(t;k\right)-g_{1}^{o}\left(0\right)\right|}^{2}}, & {\mathrm{RMSE}}_{p}\left(t\right):\mathrm{dB}=20\log_{10}{\mathrm{RMSE}}_{p}\left(t\right) \end{array}$$

(114) $$\begin{array}{ccc} {\mathrm{RMSE}}_{v}\left(t\right) & =\sqrt{\frac{1}{N_{e}}\sum_{k=1}^{N_{e}}{\left|{\hat{v}}_{g}\left(t;k\right)-v_{g,1}^{o}\left(0\right)\right|}^{2}}, & {\mathrm{RMSE}}_{v}\left(t\right):\mathrm{dB}=20\log_{10}{\mathrm{RMSE}}_{v}\left(t\right) \end{array}$$

(115) $$\begin{array}{ccc} {\mathrm{RMSE}}_{g}\left(t\right) & =\sqrt{{\mathrm{RMSE}}_{p}^{2}\left(t\right)+{\mathrm{RMSE}}_{v}^{2}\left(t\right)}, & {\mathrm{RMSE}}_{g}\left(t\right):\mathrm{dB}=20\log_{10}{\mathrm{RMSE}}_{g}\left(t\right) \end{array}$$

respectively, where ${\hat{g}}_{0}\left(t;k\right)$ denotes the initial position of the target at the *k*th experiment, $g_{1}^{o}\left(0\right)$ and $v_{1}^{o}\left(0\right)$ are constants during experiments, and ${\hat{v}}_{g}\left(t;k\right)$ denotes the estimate of the target speed at the *k*th experiment. At each random experiment, the position error and the angle measurement error are generated randomly.

### 3.1. The Convergence Curves

**Figure 3:**
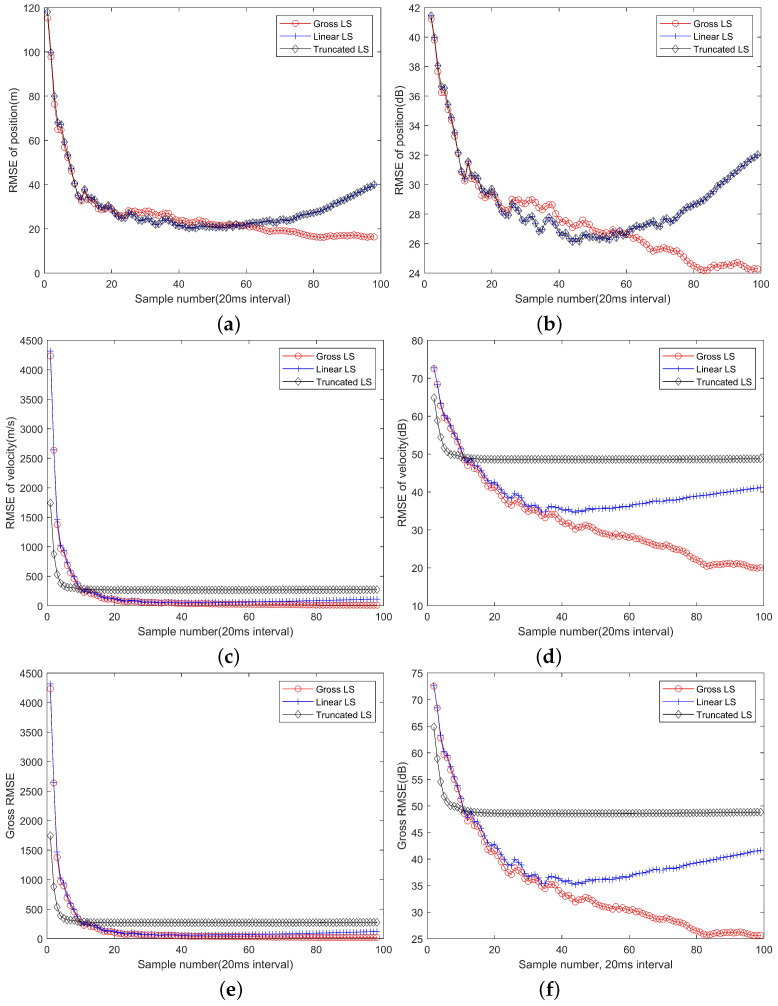
The convergence curves of the positioning errors with different numbers of observations. (**a**) The RMSE of position estimation and its dB form (**b**); (**c**) The RMSE of velocity estimation and its dB form (**d**); (**e**) The gross RMSE and its dB form (**f**).

As the number of observations increase, the localization performance will improve. Figure 3 shows the mean RMSE of position and velocity and the gross RMSE of the gross LS algorithm, the linear LS algorithm and the truncated LS algorithm.

From Figure 3a,b, it can be seen that with observations in a short while, roughly in about 0.8 s corresponding to 40 observations, all the algorithms have close gross RMSE curves. However, as more observations are available, the linear LS algorithm and the truncated LS algorithm will perform worse and the position RMSE even increase with sample number. It is a predictable result, because the linear approximations of the target and platform motion will be inaccurate gradually, resulting a deteriorated positioning performance. The gross LS algorithm will always benefit from the increase of the observations, because it does not rely on the linear approximation, and more observations will contribute more information of the target position.

From Figure 3c,d, with some initial observations, the truncated LS algorithm performs the best and the linear LS algorithm performs the worst and close to the gross LS algorithm. As more observations are involved, the truncated LS algorithm converges to a level much higher than that can be achieved by the gross LS algorithm and the linear LS algorithm. Therefore, ignoring the term ${\dot{\alpha }}_{0}$ will cause performance loss for long term observations. The gross LS algorithm is still benefitting from the increase of observations and it is slightly better than the linear LS algorithm for short term observations. The linear LS algorithm can reach a lower RMSE level but will still suffer performance degradation due to the linear model mismatch. With about 40 snapshots of observations, corresponding to 160 observations and 0.8 s period, the velocity estimation performances of two algorithms will depart.

The gross RMSE of all the algorithms are shown in Figure 3e,f, which have very close appearances to Figure 3c,d. That is because the velocity estimation errors are much greater than the positioning errors. Therefore, although the algorithm can estimate the velocity of targets, the accuracy is low due to a short observation period. In order to estimate the velocity in a higher accuracy, one needs to use observations from a longer period, which can be achieved through a filtering operation.

**Figure 4:**
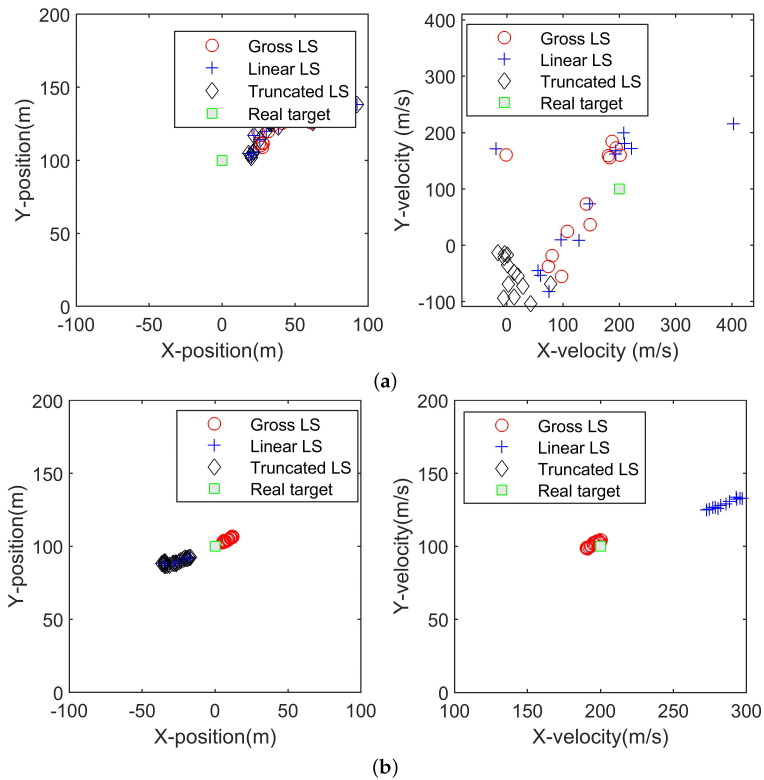
The first (**a**) and last (**b**) 20 estimates of the position and velocity of the gross LS, the linear LS and the truncated LS algorithms.

In order to show the way in which the algorithms converge to the real value, Figure 4a,b are presented to show estimated target positions and velocities at the first 20 snapshots and the latest 20 snapshots, respectively. It can be seen that with a few observations, the linear LS algorithm will converge to the real position of the target to a high accuracy. However, as more observations are available, the gross LS algorithm is closer to the real target position and the linear LS algorithm converges to other locations. Therefore, the gross LS algorithm is more robust in real applications.

### 3.2. Computation Cost

The advantage of the linear LS algorithm and the truncated LS algorithm lies in its computation cost and communication cost. In applications of the LS algorithms, the locations of the sensor will be approximated by a linear model, which is described by an initial position and a velocity term, with totally 6 parameters. Therefore, it is unnecessary to transmit all observations to the fusion center anymore and thus the communication cost will be reduced. If 100 position estimates are described by 6 parameters, the data to transmit will be reduced to 2%. Of course, there is a limit to which the data can be reduced and the limit depends on the platform speed of the sensors, the positions of sensors, the position of the target, and the periods of the observations.

**Figure 5:**
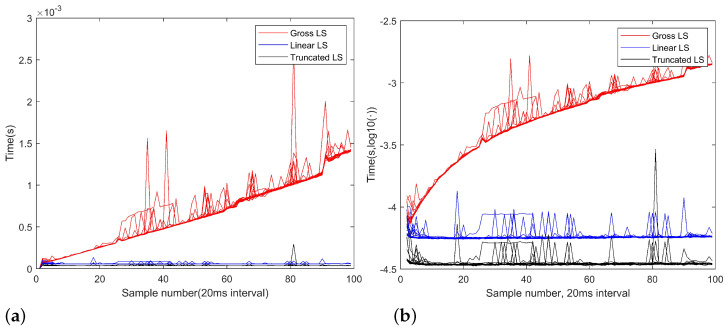
The computation time in 10 random simulations of the gross LS, linear LS and truncated LS algorithms, in scale (**a**) and dB (**b**). The program is run on a computer with an Intel™i7-10700 CPU and 16 GB memory.

The computation cost reduction, for both the linear LS algorithm and the truncated LS algorithm, stems from reduced number of multiplication and summation operations at the fusion center. The linear LS algorithm and the truncated LS algorithm can be implemented in a structure like parallel computation, namely, the linear regression of the platform position and the local DOA measures are performed at local sensors, and the fusion center just operates on the results of local sensors. To illustrate this fact, we record the computation times of the 20 random experiments for both the algorithms and show the computation times in Figure 5a,b, in scale mode and dB mode respectively. It can be seen that as the number of observations increases, the gross LS algorithm requires a longer computation time, but the linear LS algorithm and the truncated LS algorithm have much plain slopes. Meanwhile, the linear LS algorithm needs more computation cost, as a result of estimating $v_{a}$ and $v_{e}$. In fact, the computation cost of the linear LS algorithm does not vary with the sample number too much because it always computes with the same number of parameters, namely the number of sensors *N*. The computation cost increase due to more observations is imposed over local sensors now.

### 3.3. The Impact of Velocity Estimation Error

In theoretical derivations, we assumed that the velocities of sensors are estimated by position measures from a device on a platform. In practice, the platform may provide other means to measure the velocity in a higher accuracy. Meanwhile, in order to check whether the performance degradation of the linear LS algorithm in a long period is a result of inaccurate estimation of the platform velocity, we perform a simulation in a way that the estimated velocity ${\hat{v}}_{g}$ is replaced by its real value $v_{g}$. In this case, there is no velocity error and the only measurement bias is from position measurement. Note subsequently that the linear LS algorithm shares the same position estimate with the truncated LS algorithm.

**Figure 6:**
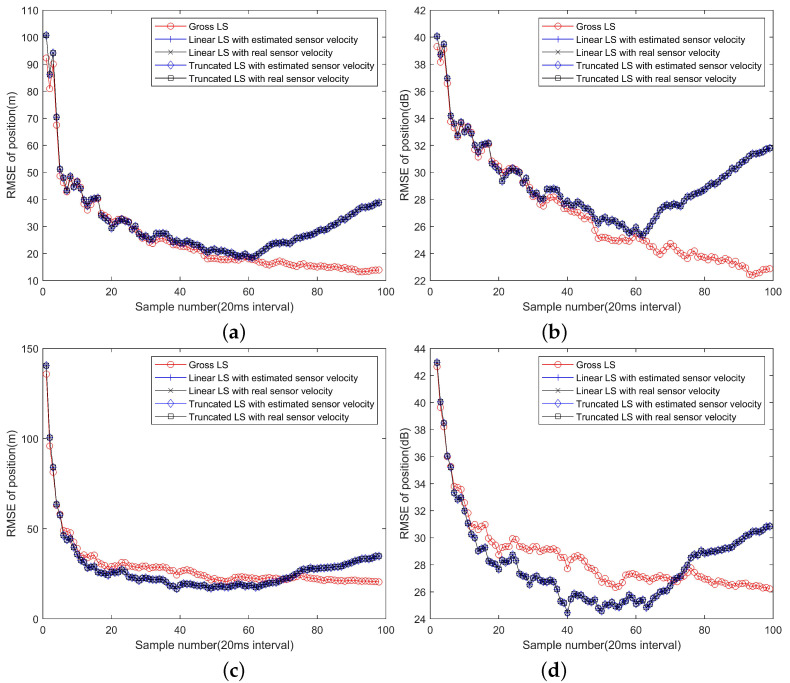
The RMSE of position with velocity estimated replaced by real velocity in scale (**a**) and dB (**b**) for $C_{s}\left(k,n\right)=I$, and in scale (**c**) and dB (**d**) for $C_{s}\left(k,n\right)=10I$.

The RMSE of position estimation is shown in Figure 6. In Figure 6a,b, the sensor location uncertainty is zero-mean normal distributed with $C_{s}\left(k,n\right)=I$. It can be clearly seen that for the linear LS algorithm, the RMSE curve with real sensor velocity is very close to the RMSE curve using estimated sensor velocity. In order to examine whether a higher position estimation error will make a difference, we make another simulation with $C_{s}\left(k,n\right)=10I$ and the results are shown in Figure 6c,d. Two RMSE curves are still very close. After some experiments with other position measurement errors, we find that the platform location and velocity regression algorithms can reach a high accuracy and thus will not cause too much performance degradation. This conclusion depends heavily on a fact that the numbers of observations under consideration is often huge according to our configurations.

In fact, from (92), the position estimate of the target does not depend on the velocity estimation of the sensor platform too much. However, there still an insignificant impact, because in our simulation configuration, the sensor location at t=0 is obtained by an interpolation operation and if the platform velocity is exactly known *a priori*, the position estimation will be more accurate.

**Figure 7:**
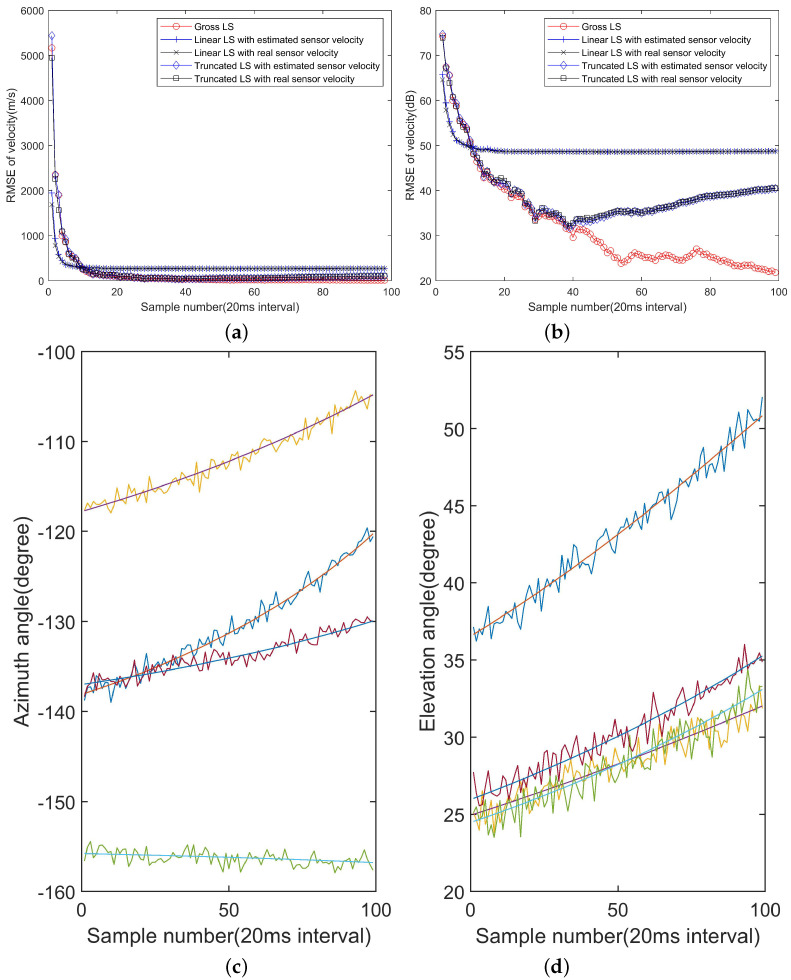
The target velocity estimation results with real and estimated sensor velocity are shown in (**a**) and (**b**) for $C_{s}\left(k,n\right)=10I$. The azimuth (**c**) and elevation (**d**) angles of the target in the four sensors.

From (105), the target velocity estimation depends on the sensor velocity more. In order the examine the impact of the sensor velocity estimation on the target velocity estimation performance, we run a simulation with $C_{s}\left(k,n\right)=10I$ and the results are shown in Figure 7a,b, in scale and dB respectively. It can be seen that it makes a little difference to use real sensor velocity instead of estimated velocity, especially in few earlier observations. As more observations are taken into account, it makes a minor different to replace by real platform velocities. That is because more observations make the velocity estimation more accurate. However, accurate sensor velocity information does not make the target velocity estimation better necessarily, and sometimes, its impact is a bit negative. As the target also moves in a constant velocity, it is reasonable to infer that the linear approximation of the signal DOA has a great impact on the target position and velocity estimation accuracy, as will be analyzed in the subsequent results.

### 3.4. Nonlinearity of the DOA Approximation

In order to examine the impact of DOA nonlinearity on the final performance, Figure 7c,d show the azimuth angles and elevation angles of the target in the four sensors. The azimuth angle and elevation angle change by about $15^{o}$ at most during 100 snapshots. Over 100 observations, corresponding to 2 s, the nonlinearity of both DOA angles becomes obvious. One should refer to explicit numerical quantities to evaluate the nonlinearity acceptable.

## 4. Conclusions

This paper studies the target position and velocity estimation problem with distributed passive sensors. The problem is formulated with distributed asynchronous sensors connected to a fusion center with communication links. We first present a gross LS algorithm that takes all angle observations from distributed sensors into account to make a LS estimation. The algorithm is simplified after some matrix manipulations, but as it needs local sensors transmitting all local observations to a fusion center, the communication cost is high. Meanwhile, the computation cost at the fusion center is also high. The communication cost is mainly a result of high-dimensional received data. In order to reduce the communication cost and computation cost, we present a linear LS algorithm that approximates local sensor locations and angle observations with linear models and then estimate target position and velocity with the parameters of the linear models. In order to simplify the velocity estimation, we also present a truncated LS algorithm that just take an average operation to estimate target velocity. In this manner, both the communication cost and the computation cost at the fusion center are reduced significantly. However, the linear LS algorithm and the truncated LS algorithm faces the model mismatching problem, namely, if the linear approximation is not accurate anymore, the performance may degrade greatly. That is a difference from the gross LS algorithm, which always benefits from more observations, as if the linear target position model holds.

The performance of the concerned algorithms is verified with numerical results. It is found that with less observations, the truncated LS algorithm performs the best. As the number of observations increase, the linear LS algorithm and the gross LS algorithm perform better. With more observations available, the linear model mismatch and then the gross LS algorithm perform the best. The gross LS algorithm always benefits from more local observations, which is a difference from the other two algorithms. The cost is a higher communication cost and a higher computation cost at fusion center. We also examined the angle distortion problem that is the only nonlinear term in the simulation configurations. Our matrix operations often make the estimation need less computation costs.

Compared to localization and tracking framework, the algorithms with velocity estimation needs a much lower rate of tracking operations, whose matrix inverse operation often need huge computation cost. Meanwhile, it can provide more accurate measures of target states and the tracking algorithm will also benefit from that. In our simulations, the sensor location error is not taken into account. In practice, this is inevitable. If the self-positioning error is non zero-mean Gaussian distributed, one may incorporate this goal in a distributed angle-only based positioning algorithm, which will be considered in our future works.

## Figures and Tables

**Table 1:** Positions and velocities of sensors and the target.

	Position (m) at *t* = 0 s	Velocity (m/s)
Sensor #1	$p_{1}^{o}\left(0\right)={\left[1000,1000,0\right]}^{T}$	${\left[-100,0,0\right]}^{T}$
Sensor #2	$p_{2}^{o}\left(0\right)={\left[1000,2000,0\right]}^{T}$	${\left[-100,-80,0\right]}^{T}$
Sensor #3	$p_{3}^{o}\left(0\right)={\left[2000,1000,0\right]}^{T}$	${\left[-100,-50,0\right]}^{T}$
Sensor #4	$p_{4}^{o}\left(0\right)={\left[1500,1500,0\right]}^{T}$	${\left[-100,-60,0\right]}^{T}$
Target #1	$g_{1}^{o}\left(0\right)={\left[0,100,1000\right]}^{T}$	${\left[200,100,0\right]}^{T}$

## Data Availability

This study did not report any data.
